# Successful Interventional Endovascular Management of Ruptured Penetrating Aortic Ulcer with Associated Enormous Right Pleural False Aneurysm

**DOI:** 10.3390/clinpract14020049

**Published:** 2024-04-17

**Authors:** Andrei Emanuel Grigorescu, Andrei Anghel, Horea Feier

**Affiliations:** 1 Department of Cardiology, “Victor Babes” University of Medicine and Pharmacy Timisoara, 300041 Timisoara, Romania; grigorescu.andrei@umft.ro (A.E.G.); horea.feier@umft.ro (H.F.); 2 Research Center of the Institute of Cardiovascular and Heart Disease of Timisoara, 300310 Timisoara, Romania; 3 Division of Cardiovascular Surgery, Institute for Cardiovascular Diseases, 300391 Timisoara, Romania; 4 Doctoral School Medicine—Pharmacy, “Victor Babes” University of Medicine and Pharmacy Timisoara, 300041 Timisoara, Romania; 5 Department of Biochemistry, “Victor Babes” University of Medicine and Pharmacy Timisoara, 300041 Timisoara, Romania

**Keywords:** penetrating aotic ulcer, false aneurysm, endovascular, stent graft

## Abstract

Penetrating aortic injuries represent critical medical emergencies that necessitate immediate intervention to prevent life-threatening consequences. When accompanied by the presence of an enormous right pleural false aneurysm, the clinical scenario becomes exceptionally rare and complex. This case report details the successful management of a patient who presented with a penetrating aortic ulcer and an extensive false aneurysm within the right pleura, employing an interdisciplinary approach involving cardiac surgeons, cardiologists, interventional cardiologists, and radiologists. The pivotal intervention involved the deployment of a covered and bare stent graft into the descending thoracic aorta to seal the aortic rupture. The patient’s clinical condition stabilized postoperatively, with no signs of recurrent hemorrhage. This case underscores the importance of rapid diagnosis, timely intervention, and the collaborative efforts of a specialized medical team in successfully managing such complex vascular injuries. Early recognition and referral to specialized centers are essential for improving patient outcomes in cases of penetrating aortic injuries with associated giant pseudoaneurysms.

## 1. Introduction

Aortic diseases encompass a broad spectrum of pathologies that pose significant challenges in contemporary medicine due to their potential for catastrophic outcomes. Among these, penetrating aortic ulcer (PAU) is a distinctive entity that has gained recognition as a critical condition deserving particular attention. It has been discovered as a separate pathology by the wide-scale use of contrast-enhanced CT and has been included in the ESC classification of acute aortic syndromes in Class IV [1]. This classification system underscores the importance of recognizing variants within aortic dissections. Notably, intramural hematoma (Class 2) and penetrating ulcer (Class 4) aortic dissections are specifically emphasized, drawing attention to the diagnostic challenges inherent in these distinct classes [2,3,4].

Though not as widely studied or recognized as classic aortic dissections or aneurysms, PAUs have garnered increasing attention in recent years due to their potentially life-threatening consequences. This pathological entity is challenging to diagnose, manage, and predict, making it a topic of growing importance in the field of vascular medicine.

Unlike classic aortic dissections, which involve the separation of aortic layers, penetrating ulcers are characterized by the erosion of the intimal layer into the media, leading to the creation of an ulceration within the aortic wall. The majority of these are asymptomatic but have the potential to evolve into more severe aortic complications, such as full-thickness aortic dissection [4,5].

We present here a case of a penetrating ulcer of the isthmic aorta, which culminated in the formation of a substantial pseudoaneurysm located within the right pleural cavity. This rare and complex clinical scenario posed both diagnostic and therapeutic challenges, necessitating a multidisciplinary approach to ensure the patient’s optimal care. The successful management of this case underscores the significance of early recognition, precise diagnostic evaluation, and timely intervention in the face of such intricate aortic pathologies.

## 2. Case Presentation

### 2.1. Patient Information

The patient at the center of this case report was a 66 year old male with limited prior medical history. From a clinical perspective, the patient’s presentation was intriguing. He sought medical attention primarily due to a hemoptysis. This raised concerns about potential underlying pulmonary or cardiovascular conditions. Notably, he had never experienced hemoptysis before, which prompted him to seek immediate medical attention. Aside from the hemoptysis, the patient was largely asymptomatic. He did not report any chest pain, shortness of breath, or palpitations. Furthermore, there were no apparent signs of hemodynamic instability, such as hypotension or tachycardia, upon initial examination.

It is noteworthy that the patient had never been admitted to a hospital previously and had no significant prior history of medical ailments. However, his lifestyle choices included a history of both alcohol consumption (5 drinks/day) and smoking (2 pack-year).

### 2.2. Clinical Findings

The patient’s journey towards the diagnosis and subsequent intervention commenced when he presented to the emergency unit, primarily complaining of several episodes of hemoptysis.

**Figure 1:**
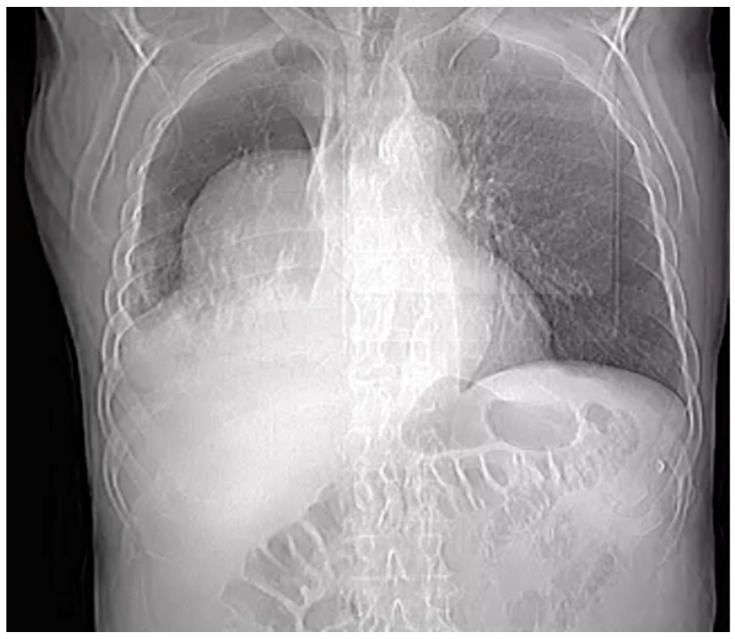
Chest X-ray.

**Figure 2:**
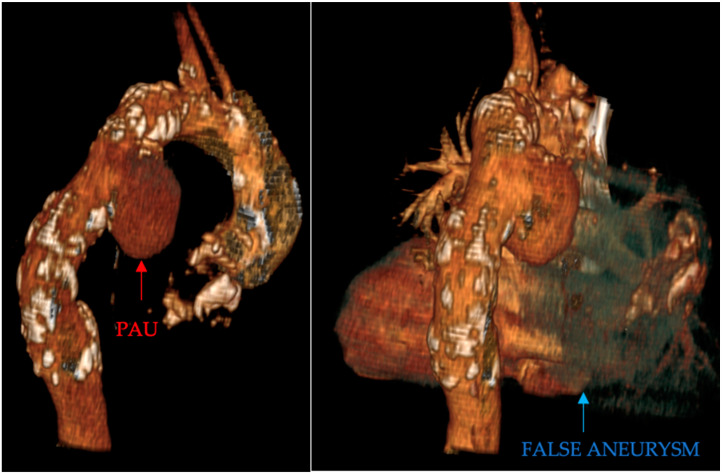
PAU and the false aneurysm.

**Figure 3:**
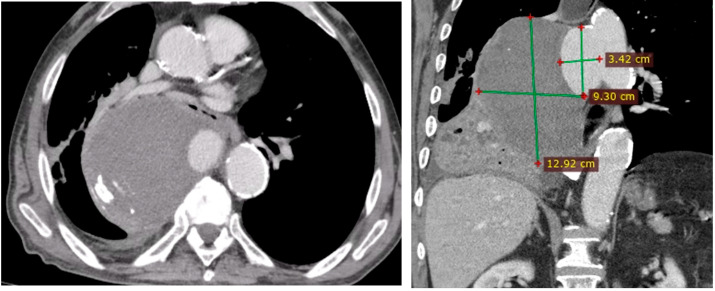
Right pleural aortic false aneurysm.

A preliminary chest X-ray was ordered as part of the initial assessment (Figure 1). The radiographic image revealed an abnormality within the right pleural cavity. To elucidate the nature of this radiographic image, a thoracic angiographic computed tomography (CT) scan was deemed essential. The key findings of the imaging study included the below:

- Penetrating aortic ulcer ruptured: As seen in Figure 2, the thoracic angio-CT scan identified a penetrating ulcer within the aortic wall, particularly located in the isthmic region, measuring approximately 4.2 cm × 3.5 cm. This was a concealed source of significant concern, as it posed an imminent risk of catastrophic hemorrhage.
- Right pleural aortic false aneurysm (Figure 3): The most striking feature was the presence of an expansive false aneurysm within the right pleural cavity, measuring approximately 12.92 cm × 9.3 cm. The size and location of this pseudoaneurysm were unprecedented, introducing a challenge in terms of diagnosis and intervention.
- Megaesophagus: Notably, the imaging findings also uncovered a megaesophagus, a condition characterized by significant dilation of the esophagus. Although not the primary focus of intervention, its presence added another layer of complexity to the clinical scenario. The etiology of the megaesophagus remained to be elucidated.
- Compression of the lung: The false aneurysm within the right pleura had exerted a significant mass effect, resulting in the compression of surrounding structures, notably the lung. This compression had led to compromised ventilation of the lung, contributing to a concerning clinical picture.
- Potential lung infection (Empyema): The compromised lung, in part due to the mass effect of the false aneurysm, exhibited signs of infection. Imaging suggested the possible development of empyema. This provided a plausible explanation for the patient’s presenting symptom of hemoptysis.

The clinical findings derived from the thoracic angio-CT scan provided critical insights into the multifaceted nature of the patient’s condition. They not only unveiled the life-threatening vascular anomalies but also shed light on associated complications, such as compromised lung function and potential infection.

### 2.3. Blood Analysis

Upon initial presentation to the emergency department (ER), the blood analysis (Table 1) unveiled a inflammatory response characterized by elevated levels of inflammatory markers (ESR = 120 mm/h). This finding was indicative of a possible underlying infection, which was later confirmed by imaging as a potential empyema within the right pleural space. A concerning hematological finding was a drop in hemoglobin levels between the time of presentation in the ER and arrival in our specialized unit. The hemoglobin levels decreased from an initial measurement of 9 g/dL in the ER to 7.9 g/dL upon arrival to our unit. This substantial reduction in hemoglobin warranted immediate attention, as it signaled acute hemorrhage.

Transfusion therapy was administered, leading to a improvement in the patient’s hemoglobin levels. Subsequent blood analyses indicated that the hemoglobin levels remained stable after the transfusion.

Empirical antibiotic therapy was initiated promptly, prior to the endovascular procedure. This approach was grounded in the principle of minimizing infection risk, particularly in the context of introducing a stent graft. The choice of antibiotics was based on broad-spectrum coverage, tailored to address both the suspected empyema and any potential infections related to the endovascular intervention.

### 2.4. Endovascular Procedure and Stent Placement

Given the critical condition of the patient, a multidisciplinary team consisting of cardiovascular surgeons, interventional cardiologists, and anesthetists was convened. After obtaining informed consent, the decision was made to proceed with an endovascular intervention.

In this case, we opted for a stent graft intervention over traditional surgery due to the urgent nature of the condition and, given the risks associated with open surgical repair, the endovascular approach was deemed the safest and most efficient treatment option.

The pivotal step in the interventional management of this case was the endovascular placement of a covered stent graft Medtronic Valiant Thoracic 36/36/150 mm. In our case, the sizing of the stent graft was meticulously conducted with the assistance of Medtronic technicians. This collaboration was instrumental in ensuring the precision and accuracy required for the procedure. We employed the Medtronic stent graft sizing chart, a tool specifically designed to facilitate optimal stent selection based on the patient’s anatomical measurements and the characteristics of the aortic pathology, in particular the size of the proximal and distal landing zones, keeping in mind the fact that for acute aortic pathologies, oversizing should be minimal to minimize the risk of rupture.

The aortic pathology extended from the distal portion of zone 3 to the proximal zone of zone 4. Given the lesion’s distribution, the decision to initiate the stent graft at landing zone 3 was deemed the safest and most efficient approach.

By commencing the stent graft in zone 3, we were able to effectively cover and seal the aortic rupture site, thereby excluding it from the circulation and preventing further hemorrhage. This strategic decision obviated the need for supplementary interventions, such as debranching procedures or extraanatomical bypasses. These additional procedures, while occasionally required in complex aortic cases, entail increased technical complexity and potential complications.

The chosen approach not only mitigated the risk of hemorrhage but also minimized the invasiveness of the intervention, resulting in a more efficient procedure. This decision was made after a thorough assessment of the patient’s anatomical suitability for the chosen landing zone, the trajectory of the stent graft, and the potential complications that could arise from alternative strategies.

To elucidate the efficacy of the deployed covered stent graft in addressing the penetrating aortic ulcer (PAU), we present a visual narrative captured through contrast-enhanced imaging.

**Figure 4:**
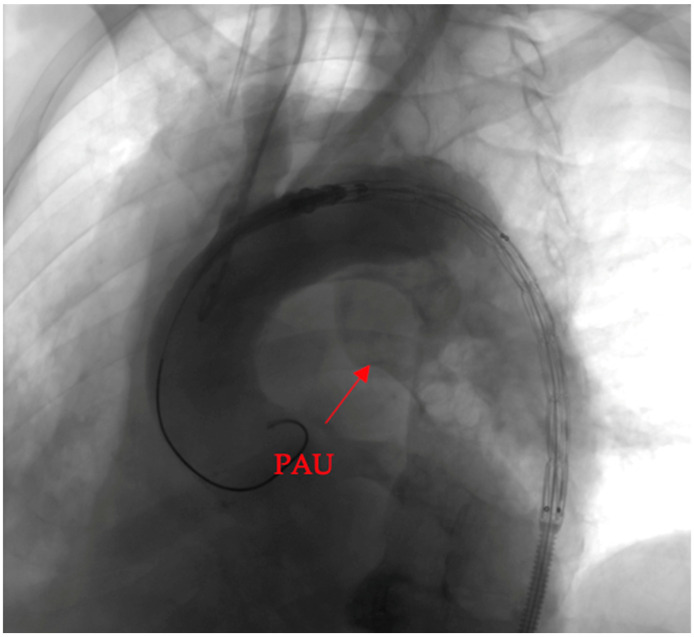
Pre-intervention assessment.

Pre-intervention assessment (Figure 4).

The initial image provides a snapshot of the aortic landscape before the intervention. Contrast material was injected to illuminate the vascular architecture, allowing for a detailed assessment of the PAU. The image showcases the breach in the aortic wall, offering a visual testament to the complexity of the lesion.

**Figure 5:**
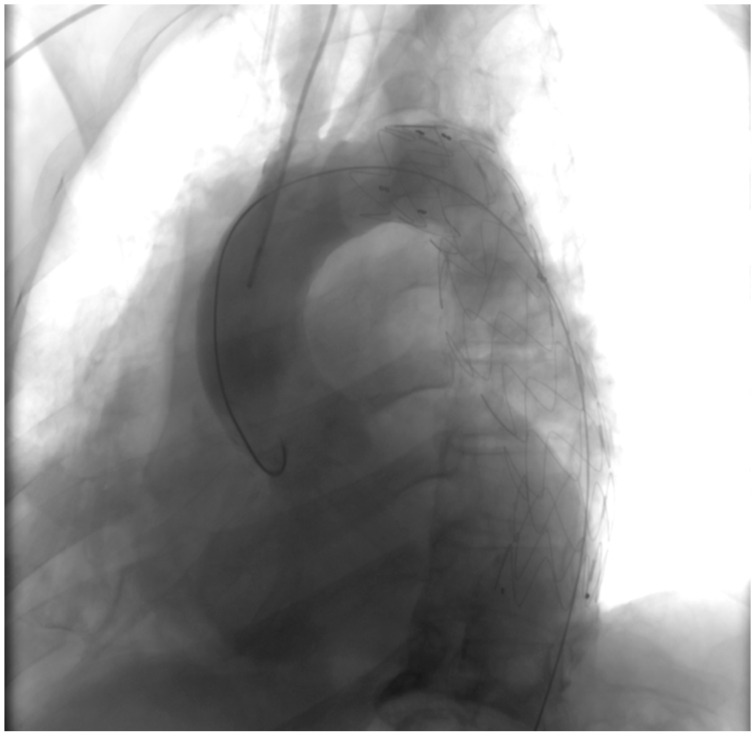
Post-intervention assessment.

Post-intervention assessment (Figure 5).

Following the deployment of the covered stent graft, contrast injection revealed a sealed and reinforced aortic wall, effectively closing the breach created by the PAU. This visual documentation serves as a compelling affirmation of the success achieved through the endovascular intervention.

The procedural aspect of our intervention, specifically the placement of the guide wire and stent, was navigated with relative ease. The total fluoroscopy time recorded was 9.5 min and the maximum skin entrance dose was measured at 239 mGy, with a total dose area product of 8803.6 uGym^2^ and a total dose of 300 mL of contrast agent.

The patient was subsequently transferred to the intensive care unit for postoperative monitoring and hemodynamic stabilization.

### 2.5. Outcome

The patient’s postoperative course was uneventful, with gradual improvement in blood pressure and respiratory status. Serial X-ray imaging showed no signs of recurrent hemorrhage or expansion of the false aneurysm. Following the successful endovascular intervention, the patient’s clinical course showed remarkable improvement. The patient was discharged from our care five days after the procedure. However, recognizing the complexity of the case, and in light of the concomitant pulmonary affection, a comprehensive post-discharge plan was meticulously devised. The patient was provided with clear recommendations for follow-up care, emphasizing the crucial involvement of both a thoracic surgeon and a pneumologist. These specialists were entrusted with the task of addressing the patient’s pulmonary condition, ensuring that the potential empyema was treated effectively, and monitoring the recovery of lung function. This multidisciplinary approach aimed to provide the patient with the most comprehensive and tailored care, addressing both the vascular and pulmonary aspects of the case to facilitate a full and sustained recovery.

The patient presented at three months after the procedure in good general condition, with no reported pulmonary distress or clinical signs of infection.

## 3. Discussion

The term Penetrating Atherosclerotic Ulcer (PAU) refers to the ulceration of an atherosclerotic plaque that breaches the intimal layer and extends into the tunica media of the aorta [6]. This clinical entity was first systematically delineated in 1986, marking a pivotal moment in the understanding of aortic pathologies [6,7]. PAUs exhibit a predilection for the descending thoracic aorta, where atherosclerosis tends to manifest more extensively compared to the ascending aorta and the aortic arch [6,8]. Despite their potential clinical significance, the true incidence of PAUs remains elusive due to their predominantly asymptomatic nature. The insidious development of these lesions often conceals their presence, making precise incidence rates challenging to ascertain. However, some studies have ventured estimates, suggesting a prevalence ranging between 2% and 11% in certain populations [6,9,10].

The occurrence of penetrating aortic injuries, characterized by the breach of the aortic wall, is frequently associated with elevated mortality rates attributed to swift blood loss. The simultaneous manifestation of an enormous false aneurysm within the right pleura represents an uncommon scenario, introducing additional layers of complexity to the clinical management of the affected patient.

Our patient’s apparent lack of significant medical history and the relatively mild nature of his presenting symptoms added a layer of complexity to the diagnostic process. Hemoptysis, while a concerning symptom, was not initially indicative of the critical underlying vascular pathology that was ultimately discovered. As such, the case underscores the importance of maintaining a high index of suspicion, even in patients with seemingly mild clinical presentations, especially in those with risk factors such as a history of tobacco and alcohol use.

Contemplating potential strategies to mitigate the quantity of bleeding in the context of the false aneurysm, a noteworthy consideration emerges—administering FVIII/VWF concentrates. This proposition stems from indirect considerations, as our patient was not subjected to specific testing for von Willebrand factor (VWF) or factor VIII deficits or any coagulopathies. However, the conceptual foundation for this proposal is rooted in the insights provided by I. Zindovic et al. [11], whose study delves into the dynamics of the von Willebrand factor within the realm of acute type A aortic dissection (ATAAD). Remarkably, ATAAD bears certain parallels with a ruptured PAU, particularly concerning the acuteness and severity of vascular compromise.

The study by Zindovic et al. [11] intriguingly suggests that the administration of an FVIII/VWF concentrate may not necessarily enhance the management of major bleeding associated with ATAAD surgery. Nevertheless, the interpretation of this information demands a nuanced lens. As we pivot to consider the applicability of this insight to our case, it becomes evident that our proposal to administer FVIII/VWF concentrates is speculative, guided by indirect considerations and the potential relevance of insights gleaned from the ATAAD study.

Crucially, our patient’s lack of specific testing for coagulation factors necessitates a cautious approach. While the cited study underscores the complexity of managing bleeding in the context of ATAAD, its direct transferability to our scenario requires careful consideration. This nuanced perspective acknowledges the limitations of extrapolating findings and reinforces the importance of tailored strategies guided by patient-specific factors and available clinical insights.

Upon scrutinizing therapeutic approaches for PAU, it becomes evident that opting for open repair can pose a multifaceted challenge. The selection of the appropriate approach hinges on several factors, most notably the precise location and clinical presentation of the PAU.

Cryopreserved homografts stand out as a particularly valuable option in cases like ours, where the risk of graft infection is heightened by comorbities, providing a significant advantage over synthetic graft materials in the context of infection risk management [12,13,14,15].

However, a critical limitation in our case was the lack of immediate access to a homograft bank, a factor that significantly constrained our treatment options.

The decision to opt for an endovascular procedure over open surgery was driven by several key factors. First, the emergency nature of the acute hemorrhage expressed by a loss in hemoglobin levels, despite the patient’s hemodynamic stability, necessitated a rapid response. TEVAR offers a more rapid means to control bleeding, a crucial factor in our decision-making process given the urgency of the situation.

The vulnerability of this patient cohort emphasizes the need to explore less invasive options to mitigate potential complications associated with traditional open repairs.

Research studies consistently demonstrate that endovascular repair is linked to lower incidences of death, stroke, and paraplegia compared with open surgical methods. For patients who are older or sustain significant injuries, TEVAR is particularly advantageous. However, for younger patients or those with aortic anatomies not conducive to TEVAR, open repair continues to be an important option. During the acute phase of treatment, TEVAR’s technical feasibility and safety profile render it an essential choice for high-risk patients [16,17,18,19,20,21].

This elevated risk profile underscores the critical need to reassess and diversify therapeutic strategies, pivoting towards approaches that could potentially mitigate such grave complications.

In the context of the presented case, the endovascular approach offered a quicker and safer solution compared to open surgery. The difficulty in approaching the aorta, the potential for erosion of nearby structures, and the heightened risk of rupturing the false aneurysm during manipulation were substantial concerns. Additionally, the structural integrity of aortic tissue in individuals with PAU may be compromised, posing greater challenge for open graft repairs [20,22]. This inherent fragility necessitates a meticulous approach to intervention, further accentuating the merit of considering endovascular repair as a preferred modality.

In navigating the complexities presented by PAUs, clinicians are compelled to consider not only the morphological characteristics of the ulcer but also its spatial orientation within the aorta. This tailored approach, aligning with international guidelines, ensures that management strategies are fine-tuned to the unique anatomical features of the lesion. Furthermore, as the understanding of PAUs continues to evolve, ongoing research and clinical insights will likely contribute to refining and expanding these guidelines, fostering an increasingly comprehensive and patient-centric approach to its management [1,20,22,23].

In instances where the PAU is located within the aortic arch or immediately distal to the left subclavian artery (LSA), open surgical intervention may become a necessity, by using a frozen elephant technique [24] or debranching and subsequent TEVAR [1,25]. However, it is worth noting that patients with PAU often remain asymptomatic, raising concerns about the potential risks associated with invasive treatments outweighing their clinical benefits. Notably, the use of general anesthesia has become the predominant choice for thoracic endovascular aortic repair (TEVAR), as it offers a secure airway and controlled ventilation, facilitates transesophageal echocardiography (TEE) monitoring, and ensures patient immobility during the procedure.

## 4. Conclusions

This case report underscores the successful interventional management of a patient presenting with a ruptured penetrating aortic ulcer and a massive right pleural pseudoaneurysm. The coordinated efforts of a multidisciplinary team were instrumental in achieving a favorable outcome. Rapid diagnosis, timely intervention, and the use of endovascular techniques can be lifesaving in such complex cases. Early recognition of these injuries and prompt referral to specialized centers equipped to handle such cases are essential to improving patient outcomes.

## Figures and Tables

**Table 1:** Blood analysis on presentation.

Blood Test	Results
Hemoglobin (g/dL)	7.9
Red cell count (10^6^/µL)	3.00
Platelets (10^3^/µL)	361
White blood cells (10^3^/µL)	8.19
ESR (mm/h)	120
PT (s)	19.8
INR	1.23

## Data Availability

Data sharing not applicable. No new data were created or analyzed in this review article.
